# Editorial: Assistive and service robots for health and home applications (RH3 - Robot Helpers in Health and Home)

**DOI:** 10.3389/fnbot.2024.1503038

**Published:** 2024-10-29

**Authors:** Paloma de la Puente, Markus Vincze, Diego Guffanti, Daniel Galan

**Affiliations:** ^1^Centre for Automation and Robotics (UPM-CSIC), Universidad Politécnica de Madrid, Madrid, Spain; ^2^Automation and Control Institute, Vienna University of Technology (TU Wien), Vienna, Austria; ^3^Universidad UTE, Quito, Ecuador

**Keywords:** assistive robots, domestic robots, health applications, perception, home navigation, grasping, human–robot interaction (HRI), system behavior

## 1 Introduction

In Choi et al. (2024), the main trends in assistive technologies for healthcare and home environments were analyzed. In that study, assistive technologies were classified into three major groups: physical aid or mobility devices, sensor and monitoring systems, and assistive robots. In an aging society, with people living longer and a lack of medical personnel, there is undoubtedly a growing interest in the development and commercialization of robotic systems that are able to provide support at healthcare facilities and home environments (Bajones et al., 2018; Keroglou et al., 2023; Silvera-Tawil, 2024). At healthcare facilities, robots improve the diagnosis and treatment of many different diseases (mental and physical), and they help professional staff be more efficient, with more time for patients. At home, they assist to prevent accidents, and they have the potential to perform different household tasks, keep the users active with cognitive and physical activities, and raise alarms if needed. Assistive robots designed for therapeutic purposes have proven useful in reducing agitation of elderly people and stress of caregivers (Kolstad et al., 2020).

One major challenge is the robustness and reliability of these robotic systems, which involve complex technologies and software, and usually operate in highly unpredictable environments (Bajones et al., 2018). Progress in fundamental problems such as object detection and recognition (Thalhammer et al., 2024), navigation (De La Puente et al., 2019a), and grasping (De La Puente et al., 2019b) is achieved every year, but more practical and robust solutions are yet to be found. Human–robot interaction (HRI) is also of utmost importance for the acceptance of such robots (Bajones et al., 2019; Fernández-Blanco Martín et al., 2023). All these topics are of course interconnected since advances in one direction may reveal and help address other kinds of issues (Bajones et al., 2018).

This Frontiers Research Topic aims at compiling a general overview of ongoing studies and recent results in these areas, specifically targeted to solve open problems for assistive and service robots in health and home applications.

## 2 Contributed articles

One significant problem related to mobile assistive robots in indoor environments is that of robust localization within a given map. Peña-Narvaez et al. present a novel strategy to enhance robot localization in intricate indoor situations by fusing classic mapping approaches with Visual Question Answering (VQA) techniques, which include the most recent developments in Monte Carlo Localization and visual language modeling. The study addresses important issues related to localization and mapping accuracy in real-world environments such as homes, offices, and medical facilities. The authors present a localization hypothesis that strengthens localization algorithms by integrating semantic insights from VQA. Experiments show that the presented methodology is better than state-of-the-art multihypothesis methods, mainly in terms of particle quality and location estimation. Accurate self-localization is achieved even in complex and dynamic situations. This approach shows a high recovery rate after intentional position perturbations, highlighting its reliability in indoor navigation.

Being able to determine its own position for navigation to known places is a key aspect for many functionalities such as transportation of different items or for cleaning tasks. However, other activities involved in support for daily living require proper detection and communication with the user. Hence, another topic of high relevance for this kind of service robots is the interaction between the human and the robot. In many cases, a successful interaction requires reliable human pose estimation algorithms. Tang and Zhao propose and evaluate an integrated machine learning model, YOLOv8-ApexNet, which shows enhanced prediction results in terms of accuracy and versatility. Their complete network architecture is able to deal with particularly challenging situations, hence opening new possibilities for improved interaction and assistance in real-world applications.

Other aspects of human–robot interaction are addresed by Rehm and Krummheuer, who investigate how socially assistive robots can improve reminding processes to support patients with memory problems in institutional care settings. The study suggests that, in addition to timely notifications, a more complex and personal approach, where the user's emotional state and cognitive abilities are considered, is needed to optimize the impact of reminding. This study contributes to social robotics by addressing personalization in companionship and care, facilitating more natural and effective interactions with elderly or vulnerable people in care facilities.

Regarding physical interaction, Ryali et al. provide interesting insights and findings from the evaluation of a wearable Exoskeletal Networks of Elastic Torque (ExoNET). Endowing healthy users with an aid for muscle activity reduction without affecting kinematics during daily living tasks, they show the great potential of this technology for simplifying and advancing rehabilitation and wellbeing of people with upper extremity mobility impairments. Limitations such as the lack of analysis for other common movements in daily activities and the need for additional subjective evaluations are also identified and clearly indicated in the study.

To carry out particularly repetitive, tiring, or damaging tasks at home, the grasping abilities play a vital role. In this regard, Cheng et al. study the advantages and limitations of shared control for grasping tasks, combining the advanced reasoning capabilities of humans with the precision of low level automatic control. This approach seems very promising for difficult tasks in domestic settings and could be used as a basis to handle irregular objects in the presence of complicated occlusions or with a limited field of view. From a different perspective, Ge et al. propose their ontology-based autonomous robot task processing framework (ARTProF) to improve an autonomous robot's performance in open-world changing environments. The framework includes a knowledge base and machine learning methods for advanced perception capabilities, manipulation based on knowledge with generalization capabilities, and a dynamic task planning algorithm to deal with unexpected situations.

Other significant challenges for health and home robots come from the integration of components toward advanced systems that provide a variety of functionalities to support the users. The study by  Mora et al. describes the ADAM robot, designed to assist elderly people by performing household tasks such as cleaning and picking up objects. This setup contributes to improving the autonomy of older people in the home. ADAM, equipped with a manipulation system consisting of two robotic arms and autonomous navigation, also learns from human interactions. This study contributes significantly to social robotics by integrating physical assistance functions with social interaction capabilities, reducing loneliness, and providing better care for older people, enhancing their independence and wellbeing.

Finally, the study by Sierra M. et al. addresses the growing healthcare need of an aging population toward aids for assisted mobility. While there exist several walking aids, there is a lack of assistive technologies with social capabilities that are cost-effective, user-friendly, and readily adopted. In this study, a socially assistive walker is designed to provide physical and cognitive support in activities of daily living for older adults. Physical and cognitive support is provided by the walker's structure, sensors, and feedback interfaces to assist users during daily activities while navigating the environment safely and efficiently. The walker presents two levels of social interaction with low and high interaction. An evaluation with 14 adults showed that the walker is easy to use regardless of the interaction mode and the majority of users expressing a preference for the version featuring embodiment, verbal feedback, and more proactive cues.

## 3 Conclusion

This Frontiers Research Topic integrates very different aspects of robotics for health and home applications, showing the great variety of challenges that are involved for fully operational multi-function solutions. From robot localization and human pose estimation to grasping, mental and physical HRI and integrated robotic devices for more advanced support, novel ideas are presented and evaluated.

From the Research Topic contributions, we would like to highlight some key areas for ongoing research. In mobile robot navigation for domestic environments, achieving more accurate localization in long and large symmetric spaces and the ability to relocalize are important capabilities that will attract more attention. In this field, and also related to HRI, there is a current trend toward the integration and exploitation of semantic information. Regarding human pose estimation, the next steps should address the problem of difficult occlusions, abnormal poses, and special motions.

Future studies should also focus on more empirical testing with real users in real environments, both for individual functionalities and for multi-purpose robots and assistive devices. The benefits of shared control for performing difficult tasks should also be further studied, integrated and evaluated. The ethical implications of the development and adoption of assistive and medical robots should be further analyzed.
